# PfAP2-G2 Is Associated to Production and Maturation of Gametocytes in *Plasmodium falciparum via* Regulating the Expression of *PfMDV-1*

**DOI:** 10.3389/fmicb.2020.631444

**Published:** 2021-01-18

**Authors:** Yaozheng Xu, Dan Qiao, Yuhao Wen, Yifei Bi, Yuxi Chen, Zhenghui Huang, Liwang Cui, Jian Guo, Yaming Cao

**Affiliations:** ^1^Department of Immunology, College of Basic Medical Sciences, China Medical University, Shenyang, China; ^2^Department of Laboratory Medicine, Ruijin Hospital, Shanghai Jiao Tong University School of Medicine, Shanghai, China; ^3^Institut Pasteur of Shanghai, Chinese Academy of Sciences, Shanghai, China; ^4^Philosophy, Politics and Economics Department, University College London, London, United Kingdom; ^5^Department of Internal Medicine, Morsani College of Medicine, University of South Florida, Tampa, FL, United States; ^6^Department of Laboratory Medicine, Shanghai East Hospital, Tongji University School of Medicine, Shanghai, China

**Keywords:** malaria, *Plasmodium falciparum*, gametocytes, sexual development, transcriptional factor

## Abstract

Gametocyte is the sole form of the *Plasmodium falciparum* which is transmissible to the mosquito vector. Here, we report that an Apicomplexan Apetala2 (ApiAP2) family transcription factor, PfAP2-G2 (Pf3D7_1408200), plays a role in the development of gametocytes in *P. falciparum* by regulating the expression of *PfMDV-1 (Pf3D7_1216500)*. Reverse transcriptase-quantitative PCR (RT-qPCR) analysis showed that *PfAP2-G2* was highly expressed in the ring stage. Indirect immunofluorescence assay showed nuclear localization of PfAP2-G2 in asexual stages. The knockout of *PfAP2-G2* led to a ~95% decrease in the number of mature gametocytes with a more substantial influence on the production and maturation of the male gametocytes, resulting in a higher female/male gametocyte ratio. To test the mechanism of this phenotype, RNA-seq and RT-qPCR showed that disruption of *PfAP2-G2* led to the down-regulation of male development gene-1 (*PfMDV-1*) in asexual stages. We further found that PfAP2-G2 was enriched at the transcriptional start site (TSS) of *PfMDV-1* by chromatin immunoprecipitation and qPCR assay in both ring stage and schizont stage, which demonstrated that *PfMDV-1* is one of the targets of PfAP2-G2. In addition, RT-qPCR also showed that PfAP2-G (Pf3D7_1222600), the master regulator for sexual commitment, was also down-regulated in the PfAP2-G2 knockout parasites in the schizont stage, but no change in the ring stage. This phenomenon suggested that PfAP2-G2 played a role at the asexual stage for the development of parasite gametocytes and warrants further investigations in regulatory pathways of PfAP2-G2.

## Introduction

Malaria is a vector-borne disease caused by protozoan parasites of the genus *Plasmodium*. Nearly half of the world's population is at risk of malaria and hundreds of millions of people suffer from the disease each year. Of the five species that infect humans, *Plasmodium falciparum* is the most lethal. The complete lifecycle of *P. falciparum* includes developmental stages that occur in a mosquito vector, the human liver, and human blood (Ciuffreda et al., 2020). Though the symptoms of the disease are associated with the asexual growth of the parasites in the human blood, the parasites must undergo sexual differentiation to form female and male gametocytes, so that the mature gametocytes in the human peripheral blood can be taken up by the mosquito and transmitted. Therefore, gametocyte is an attractive target for eliminating malaria (Henry et al., 2019; Amoah et al., 2020; Guiguemde et al., 2020).

The Apicomplexan Apetala2 (ApiAP2) family is the sole family of sequence-specific transcriptional factors that have been identified in *Plasmodium* species, with each protein containing one to three copies of the AP2 integrase DNA binding domains (Balaji et al., 2005; De Silva et al., 2008). The DNA-binding specificities of orthologous pairs of AP2 domains are fundamentally conserved, and the timing of orthologous ApiAP2 protein expression is very similar, although the putative target genes of orthologous ApiAP2s are sometimes highly divergent (Oberstaller et al., 2014; Martins et al., 2017). So far, five ApiAP2 proteins have been identified as key stage-specific regulators in the rodent malaria parasite *Plasmodium berghei* (Yuda et al., 2009, 2010; Iwanaga et al., 2012; Kafsack et al., 2014; Josling et al., 2018). Though seven AP2s have been functionally characterized at the cellular level in *P. falciparum*, only PfAP2-G is associated with stage specificity and has been identified as a master regulator of sexual commitment (Flueck et al., 2010; Han et al., 2014; Kafsack et al., 2014; Henriques et al., 2015; Martins et al., 2017; Santos et al., 2017; Sierra-Miranda et al., 2017). Campbell et al. (2010) characterized the DNA binding motifs for 27 distinct AP2 members which were predicted bioinformatically in *P. falciparum*. In addition to regulating target genes, AP2 members may also regulate their own expression (Bischoff and Vaquero, 2010; Campbell et al., 2010). Previous studies of AP2-G2 in *P. berghei* demonstrated that PbAP2-G2 disruptants almost completely lacked mature gametocytes, suggesting that this transcriptional factor played a critical role in the maturation of gametocytes (Sinha et al., 2014; Yuda et al., 2015; Modrzynska et al., 2017). In addition, PbAP2-G2 targeted about 1,500 genes mostly required for asexual replication, repressing them before the commitment to sexual differentiation, which was thought to induce genome-wide gene repression in both asexual blood stages and gametocytes (Sinha et al., 2014; Martins et al., 2017). Recently, Singh et al. (2020) reported that PfAP2-G2 played a critical role in the maturation of gametocytes and bound to the promoters and gene body of a wide array of genes, acting as a repressor.

In this study, we report that PfAP2-G2 plays a role in the production and maturation of gametocytes. First, we detected that *PfAP2-G2* was highly expressed in the ring stage and localized in the nucleus of the parasites. Subsequently, we found that disruption of *PfAP2-G2* almost completely arrested gametocyte development, especially the male gametocytes. We further investigated the expression profile of this transcriptional factor in the asexual stage, and we showed that the expression levels of many genes were decreased significantly in the PfAP2-G2 disruptants, especially the male development gene 1 (*MDV-1*), which is an essential regulator in the development of male gametocytes. Chromatin immunoprecipitation and quantitative PCR (CHIP-qPCR) assay showed that PfAP2-G2 was enriched at the transcriptional start site (TSS) of *PfMDV-1* in the asexual stage. This study implies an important role for PfAP2-G2 in the development of gametocytes in *P. falciparum*, possibly through regulating *PfMDV-1* and other gametocyte-specific genes in the asexual stage.

## Methods

### Parasites Culture and Transfection

*P. falciparum NF54* parasites were cultured and synchronized following the standard protocols (Trager and Jensen, 2005). Red blood cells were transfected with 100 μg of each of the plasmids PL6CS-hDHFR-AP2-G2 and PL6CS-hDHFR-AP2-G2(-) with pUF1-BSD-Cas9 by electroporation. Schizont-stage parasites were synchronized with Percoll. Transfected parasites were subjected to selection with the WR99210 (Jacobus Pharmaceuticals) and blasticidin (BSD) (Thermo) drugs, which respectively targeted human dihydrofolate reductase and blasticidin S deaminase, to select parasites carrying both pUF1-BSD-Cas9 and PL6CS-hDHFR-AP2-G2(-) or both pUF1-BSD-Cas9 and PL6CS-hDHFR-AP2-G2. After resistant parasites emerged, genomic DNA was extracted for PCR validation, and parasites were cloned by limiting dilution. The knockout and tagging of *PfAP2-G2* were carried out separately, using the CRISPR/Cas9 system, and was confirmed by PCR. For tagging, the coding sequences of 3Ty1-3Flag was fused to the C terminus of *PfAP2-G2*. The selected tagged parasite was confirmed by PCR with genomic DNA and primer pairs P1/P2, by sequencing of PCR products, and by Western blotting. For gene knockout, the entire 397 bp functional domain (4,645 bp away from ATG to 5042 bp) was replaced by the 5′-UTR-hdhfr-3′-UTR drug cassette. PCR was performed with genomic DNA as templates, and primer pairs P3/P4. The selected knockout parasite was confirmed by sequencing of the PCR products. All primers used are listed in Supplementary Table 1.

### Gametocytes Induction

To compare gametocyte development between the NF54 and the *PfAP2-G2(-)* parasites, tightly synchronized schizont stage cultures were set to a parasitemia of 0.3 with 6% hematocrit and cultivated in 15 ml of the complete medium at 37°C. We regard this day as the first day post-gametocyte induction. The medium was exchanged daily and increased to 25 ml until the parasitemia up to 5%. Cultures were maintained in culture medium for 12 days. The asexual parasites were eliminated by N-acetyl-D-glucosamine (Sangon Biotech) from day 6 post-gametocyte induction when both the NF54 and the *PfAP2-G2(-)* parasites were at the ring stage and the parasitemia reached about 13%. The cultures of NF54 and the *PfAP2-G2(-)* parasites were treated with N-acetyl-D-glucosamine for 7 days until day 12 post-gametocyte induction. Samples were taken every 24 h starting from day 2 post-gametocyte induction for Giemsa smear preparation.

### Indirect Immunofluorescence Assay (IFA)

IFAs were performed as previously described (Zhang et al., 2014). The early differences in gametocyte formation and sex ratio between the *PfAP2-G2(-)* parasites and the NF54 parasites were detected by IFA. Briefly, on day 6 post-gametocytes induction, parasites were collected and adsorbed on slides treated by poly-L-lysine for 10 min. The slides were washed twice with phosphate-buffered saline (PBS, pH 7.0). Then, the parasites were fixed with 4% paraformaldehyde and 0.0075% glutaraldehyde in PBS for 30 min. Following another PBS wash, parasites were permeabilized with 0.25% Triton X-100 in PBS for 15 min and then treated with 50 mM NH_4_Cl in PBS for 10 min. After washing the slides with PBS, the parasites were blocked with 3% BSA-PBS for 2 h. Following a PBS wash, rabbit anti-Pfs16 antibody (diluted to 1:250 in 3% BSA-PBS, BEI Resources) and mouse anti-α-tubulin antibody (diluted to 1:1,000 in 3% BSA-PBS, Sigma) were added respectively and the mixtures were incubated at 4°C overnight. After washing the slides with PBS, fluorescein isothiocyanate (FITC)-conjugated goat anti-rabbit immunoglobulin antibody and FITC-conjugated goat anti-mouse immunoglobulin antibody (Thermo) diluted to 1:2,000 in 3% BSA-PBS were added respectively and the mixture was incubated for 1 h. Parasite nuclei were counterstained with DAPI-Aqueous (Abcam). IFA slides were observed under an Olympus FV1200 microscope. The location of PfAP2-G2 by mouse anti-Flag antibody (diluted to 1:1,000 in 3% BSA-PBS, Sigma) was identified by IFA as mixed asexual parasites using the same procedure as described above.

### Western Blot Analysis

Parasites pellets were released from infected red blood cells with 0.15% saponin and then lysed in PBS containing 1% SDS and protease inhibitor. After centrifugation, the supernatant was mixed with SDS-PAGE loading buffer, separated in SDS-PAGE, and transferred to a PVDF membrane. The membrane was blocked with 5% non-fat milk in Tris-buffered saline with 0.1% Tween 20 (TBS-T) at room temperature for 2 h and probed with mouse anti-Flag (diluted to 1:1,000 in 5% non-fat milk, Sigma) and rabbit anti-H3 (diluted to 1:2,000 in 5% non-fat milk, Abcam), respectively. After three washes with TBS-T, the membranes were incubated with the secondary antibodies conjugated to HRP (Jackson Immuno Research Laboratories). Chemiluminescent HRP substrate Immobilon Western kit (Millipore) was used to develop the blots.

### RNA-seq and RT-qPCR

Total RNA was extracted from tightly synchronized *PfAP2-G2(-)* parasites and NF54 parasites at both ring stage and schizont stage using Trizol (Life Technologies) according to the manufacturer's instructions. Total RNA was dissolved in RNase-free water for downstream uses. The mRNA-seq library was constructed by KAPA Stranded mRNA-seq Kit (KAPA Biosystems, KK8420) according to the manufacturer's instructions. Barcoded libraries were pooled and sequenced with the read lengths of 150 nt from both ends on the HiSeq Ten machine (Illumina, CA). For RT-qPCR, cDNA was synthesized using the FastQuant RT kit (Tiangen). qPCR was performed on the ABI 7900 system with an initial denaturing at 95°C for 5 min, followed by 40 cycles of 10 s at 95°C, 20 s at 50°C and 30 s at 60°C. The housekeeping gene serine-tRNA ligase (Pf3D7_0717700) was used as internal control. RT-qPCR for detecting the expression level of PfAP2-G2 used the same procedure as described above. RT-qPCR was repeated three times and RNA-seq was repeated twice. Primers for RT-qPCR are shown in Supplementary Table 1.

### Chromatin Immunoprecipitation (ChIP)

The ChIP assay was performed as previously described (Chookajorn et al., 2007) using synchronized ring stage and schizont stage PfAP2-G2-3Ty1-3Flag parasites. Briefly, iRBCs were fixed with 1% formaldehyde solution (Thermo) at room temperature for 20 min and stopped by the addition of 125mM glycine at room temperature for another 5 min. Parasites were released by using 0.15% saponin. Chromatin was sheared to a size range between 200 and 300 bp by Bioruptor UCD-200 (Diagenode). Antibodies against Flag (Sigma) was used in this assay. DNA was extracted by phenol-chloroform. The ChIP-qPCR assay used the same aliquot of immunoprecipitated DNA and was detected on the ABI 7900 system. Primers for CHIP-qPCR are shown in Supplementary Table 1.

### RNA-seq Data Analysis

The raw data were filtered by trim-galore (version 0.4.4) to remove adapters, short reads and the other low quantity reads with cutoffs (reads quantity ≤ 15 and read length ≤ 15 nt). The paired and unpaired (reads length≥18) reads were aligned to the *P. falciparum* v36 genome using HISAT2 (version 2.1.0) with default parameters (Kim et al., 2015). Alignment files were converted to “BAM” format and merged using SAMtools. The PCR deduplicates were removed using Picard (version 2.18.6) with default parameters and calculated Transcripts Per Million (TPM) using Stringtie (version 1.3.4, parameters “-e –rf -j 1”) (Pertea et al., 2015). Two biological replicates for each sample were counted and mapped reads for all the genes using Stringtie and conducted differential expression analysis using DESeq2 (version 1.16.1) (Feng et al., 2012) in R platform. Finally, each gene in the treatment group against the control group was presented as the volcano plot using the ggplot2 package (version 3.1.1) (Ginestet, 2011) in R. Genes whose TPM was <10 in any replicate of a group were considered as low expression level and genes that were low expression level in both treatment and control group were considered as unchanged in mRNA level. Up-regulated and down-regulated genes were defined using the following cutoff: | log_2_ fold change | > 1.

## Results

### *PfAP2-G2* Is Highly Expressed in the Ring Stage and Localized in the Nuclei of the Parasites

**Figure 1:**
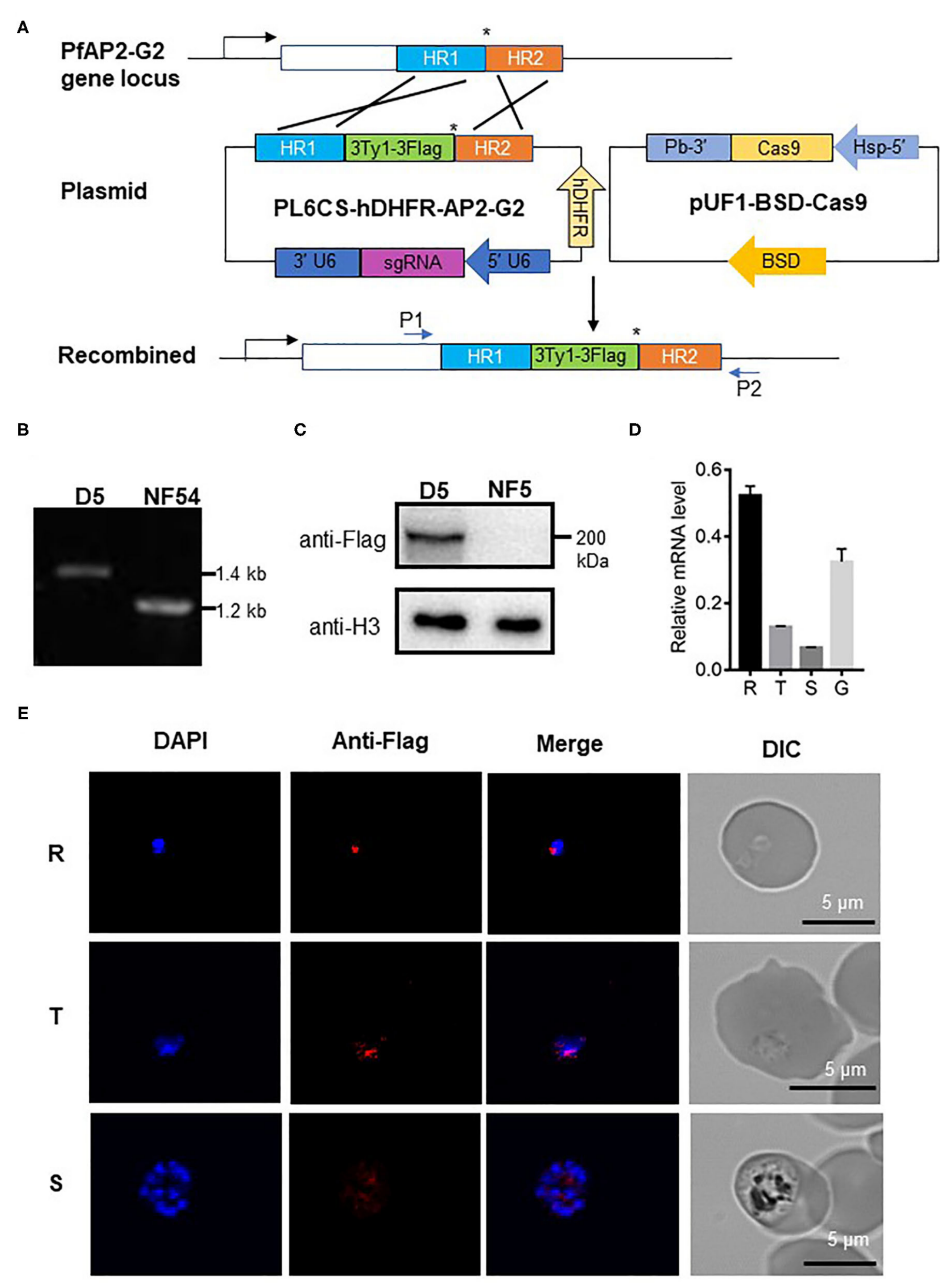
Expression of *PfAP2-G2* in *P. falciparum* development. **(A)** Schematic representation of the generation of transgenic PfAP2-G2-3Ty1-3Flag line. Co-transfection of the plasmid pUF1-BSD-Cas9 with PL6CS-hDHFR-AP2-G2 leads to the integration of the PfAP2-G2-3Ty1-3Flag containing cassette into the endogenous *PfAP2-G2* locus. HR1 and HR2 are the two homologous arms used for recombination. The asterisk (*)indicates the stop codon. P1 and P2 are primers used to verify plasmid integration. **(B)** PCR analysis of genomic DNA from the NF54 parasites and the PfAP2-G2-3Ty1-3Flag clone D5. Predicted DNA fragment sizes with P1 and P2 are 1,423 bp from D5 parasites and 1,217 bp from the NF54 parasites. **(C)** Western blot analysis of D5 parasites. Proteins were separated by 6% SDS-PAGE and probed with the mouse anti-Flag antibody and against *P. falciparum* H3 as a protein loading control. **(D)** RT-qPCR analysis of *PfAP2-G2* expression in the ring (14~16 h) (R), trophozoite (34~36h) (T), schizont (44~46 h) (S) stages and gametocyte stage IV~V(G). Primers for RT-qPCR are shown in Supplementary Table 1. The experiment was repeated three times. Error bars indicate standard deviation (SD). **(E)** IFA analysis of PfAP2-G2 during asexual parasite development. PfAP2-G2 localization was identified by using the anti-Flag antibody (red), while nuclei were counterstained with DAPI. Scale bars, 5 μm.

To explore the expression and localization of PfAP2-G2, we established parasites line with *PfAP2-G2* tagged at its C terminus with the 3Ty1-3Flag in the NF54 parasites using the CRISPR/Cas9 system (Figure 1A). The selected parasite clone (named D5) was confirmed by PCR and DNA sequencing of the PCR product (Figure 1B), as well as by Western blot to detect the tagged protein (Figure 1C). We found that tagging of *PfAP2-G2* had no effect on asexual and sexual development of the parasites (Supplementary Figure 1). We found that *PfAP2-G2* was expressed throughout the intraerythrocytic development cycle and gametocyte stage IV~V via RT-qPCR with the expression level reaching the highest in the ring stage and lowest in the schizont stage (Figure 1D). IFA of fixed asexual parasites detected that at the ring stage *PfAP2-G2* was shown as a single punctum at the nuclear periphery demarcated by DAPI staining, while in the trophozoite stage, it diffused and overlapped more extensively with the nucleus. However, the anti-Flag staining became much fainter at the schizont stage and the more prominent foci did not overlap with the DAPI staining (Figure 1E).

### *PfAP2-G2* Is Important for Gametocyte Development, Especially for Male Gametocytes

**Figure 2:**
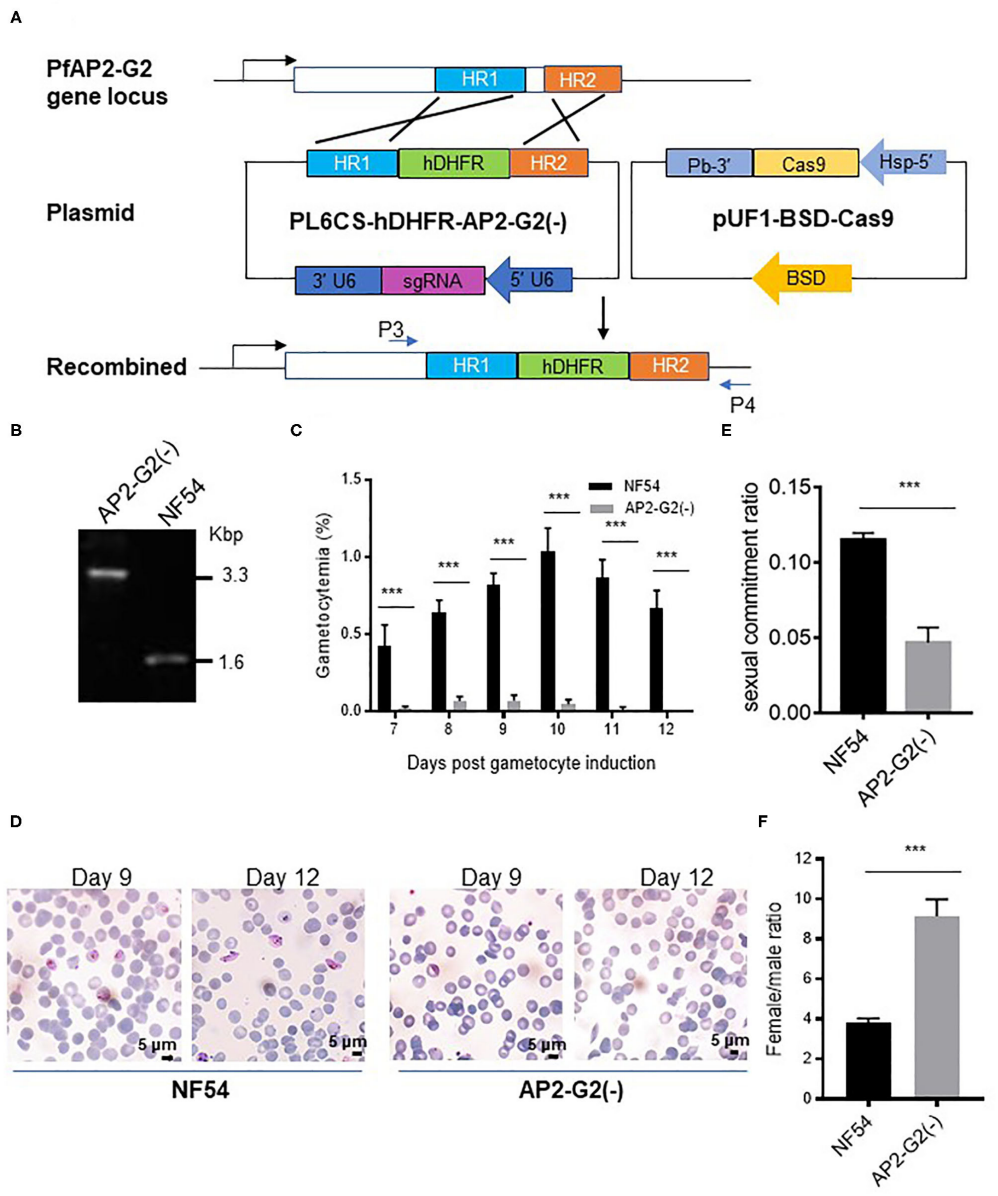
Phenotypic analysis of the *PfAP2-G2(-)* parasites. **(A)** Schematic representation of the generation of transgenic *PfAP2-G2* knockout line. Co-transfection of the plasmid pUF1-BSD-Cas9 with PL6CS-hDHFR-AP2-G2(-) leads to knockout of PfAP2-G2 structural domain in the endogenous locus. HR1 and HR2 are the two homologous arms used for recombination. P3 and P4 are primers used to verify the deletion in *PfAP2-G2*. **(B)** PCR analysis of genomic DNA from the NF54 parasites and the *PfAP2-G2(-)* clone. Predicted DNA fragment sizes with P3 and P4 are 3,330 bp from the *PfAP2-G2(-)* parasites and 1,590 bp from the NF54 parasites. **(C)** Daily gametocytemia after day 6 post-gametocyte induction. Results are the mean of three biological replicates (10,000 RBCs counted per sample). **(D)** Giemsa-stained blood smears of NF54 (left) and the *PfAP2-G2(-)* parasites (right) on days 9 and 12 to illustrate the gross morphology of the gametocytes and their disappearance in day 12 culture in the *PfAP2-G2(-)* parasites. Scale bars, 5 μm. **(E)** Sexual commitment ratio on day 6 post-induction. Results are the mean of three biological replicates (1000 infected RBCs counted per sample). **(F)** Female/male gametocyte ratio on day 6 post-induction. Results are the mean of three biological replicates (1000 infected RBCs counted per sample). Sex was differentiated based on the staining of all gametocytes by the anti-Pfs16 antibodies and strong fluorescence of male gametocytes by the anti-α-tubulin II IFA. The experiment was repeated three times. All error bars indicate SD. ***P < 0.001.

To determine the role of PfAP2-G2 during *P. falciparum* development, we replaced a coding region of *PfAP2-G2* with the hdhfr cassette using the CRISPR/Cas9 system (Figure 2A). The knockout strain [named *PfAP2-G2(-)*] was obtained after transfection and cloning and was confirmed by PCR and DNA sequencing of the PCR product (Figure 2B). Phenotypic analysis of *PfAP2-G2(-)* parasites was compared with the NF54 strain. Although the *PfAP2-G2(-)* parasites and the NF54 control showed no significant difference in daily asexual parasitemia (data not shown), there was a ~95% reduction in gametocytemia in the *PfAP2-G2(-)* parasites at day 9 after post-gametocyte induction (Figure 2C). However, gametocytes produced by *PfAP2-G2(-)* parasites retained normal morphology observed under a light microscope on day 9 post-gametocyte induction (Figure 2D). However, on day 12 post-gametocyte induction when mature gametocytes were visible in the NF54 parasites, gametocytes could not be observed in the *PfAP2-G2(-)* parasites (Figure 2D). On day 6 post-gametocyte induction, when the gametocytes were at stage I, we found that deletion of *PfAP2-G2* affected sexual commitment as determined by staining with anti-Pfs16, with the *PfAP2-G2(-)* parasites having lower sexual commitment (4.7%) than the NF54 parasites (11.5%) (Figure 2E). Simultaneously, we also found that deletion of *PfAP2-G2* had a substantial impact on the sex ratio as determined by staining with anti-α-tubulin-II antibodies, with the *PfAP2-G2(-)* parasites having significantly higher female/male gametocyte ratio (~9.1:1) than the NF54 parasites (~3.7:1) (Figure 2F). It is worth mentioning that α-tubulin II expressed normally in the *PfAP2-G2(-)* parasites (Supplementary Tables 2, 3) and anti-α-tubulin II reacted with both sexes, but the different intensities allowed differentiation of the male gametocytes (Schwank et al., 2010) (Supplementary Figure 2). Altogether, these phenotypic analyses showed more severe developmental defects in male gametocytogenesis in the *PfAP2-G2(-)* parasites.

### Downregulation of *PfMDV-1* in AP2-G2(-) Parasites

**Figure 3:**
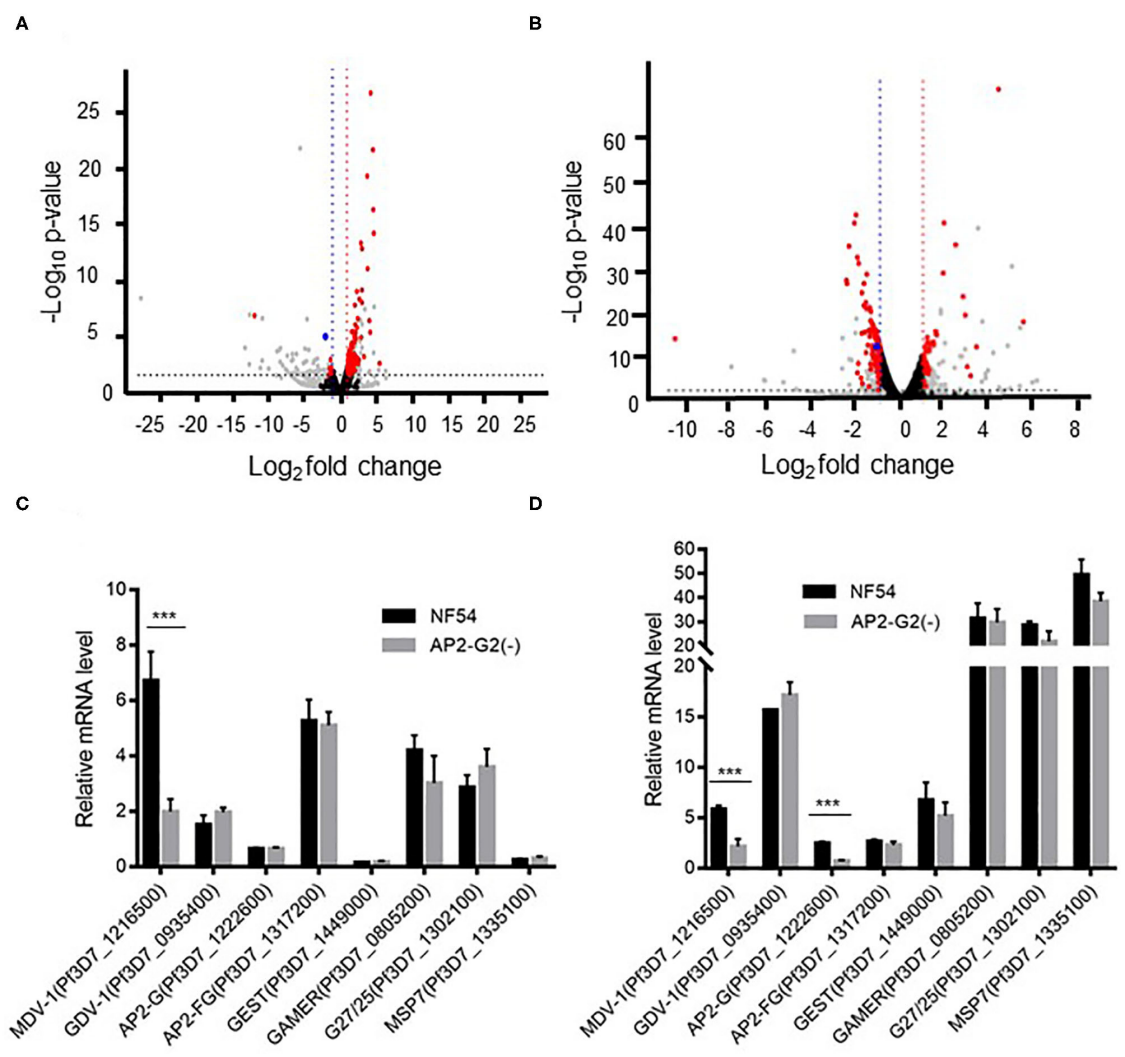
Transcriptomic analysis of NF54 and the *PfAP2-G2(-)* parasites by RNA-seq. **(A,B)** Volcano plot of log_2_ fold change (x axis) in gene expression of *PfAP2-G2(-)* parasites vs. NF54 parasites in ring stage **(A)** and schizont stage **(B)** against the significance of change shown as the –log_10_ *p*-value (y axis). Red dots indicate genes with significant changes in expression level. Blue dots indicate the *PfMDV-1* gene. Gray dots indicate genes whose TPM was <10. Black dots indicate genes whose p-value was more than 0.05 or | log_2_ fold change | was <1 **(C,D)** RT-qPCR validation of the RNA-seq data using selected genes essential for gametocyte development at the ring **(C)** and schizont **(D)** stages. A gene (*MSP7*), which is unrelated to gametocyte development, was used as a negative control. Primers for RT-qPCR are shown in Supplementary Table 1. The experiment was repeated three times. All error bars indicate SD. ***P < 0.001.

To determine the effect of PfAP2-G2 deletion on gene expression, we performed RNA-seq analysis and compared the transcriptomes of the NF54 with *PfAP2-G2(-)* parasites at the ring stage and schizont stage. Compared with the NF54 parasites, *PfAP2-G2* deletion resulted in significantly more genes up-regulated (113 genes) than down-regulated (12 genes) in the ring stage (Figure 3A, Supplementary Table 2). In the schizont stage, however, 41 and 102 genes were up-regulated and down-regulated, respectively in *PfAP2-G2(-)* parasites (Figure 3B, Supplementary Table 3). GO term analysis did not identify specific clusters of genes with altered expression. However, we found that the expression level of *PfMDV-1*, which is essential to the development of male gametocytes, was significantly decreased in both the ring and schizont stages. The expression level of *PfAP2-G*, the master regulator of sexual commitment, was also decreased in the schizont stage, but not in the ring stage. We selected *PfMDV-1, PfAP2-G*, and five other genes related to gametocyte development for RT-qPCR verification, and the results were consistent with those from RNA-seq analysis (Figures 3C,D). These results suggested that PfAP2-G2 played a role in the development of gametocytes by regulating the expression of *PfMDV-1* as well as *PfAP2-G* in the asexual stage.

### *PfMDV-1* Is a Target of PfAP2-G2

**Figure 4:**
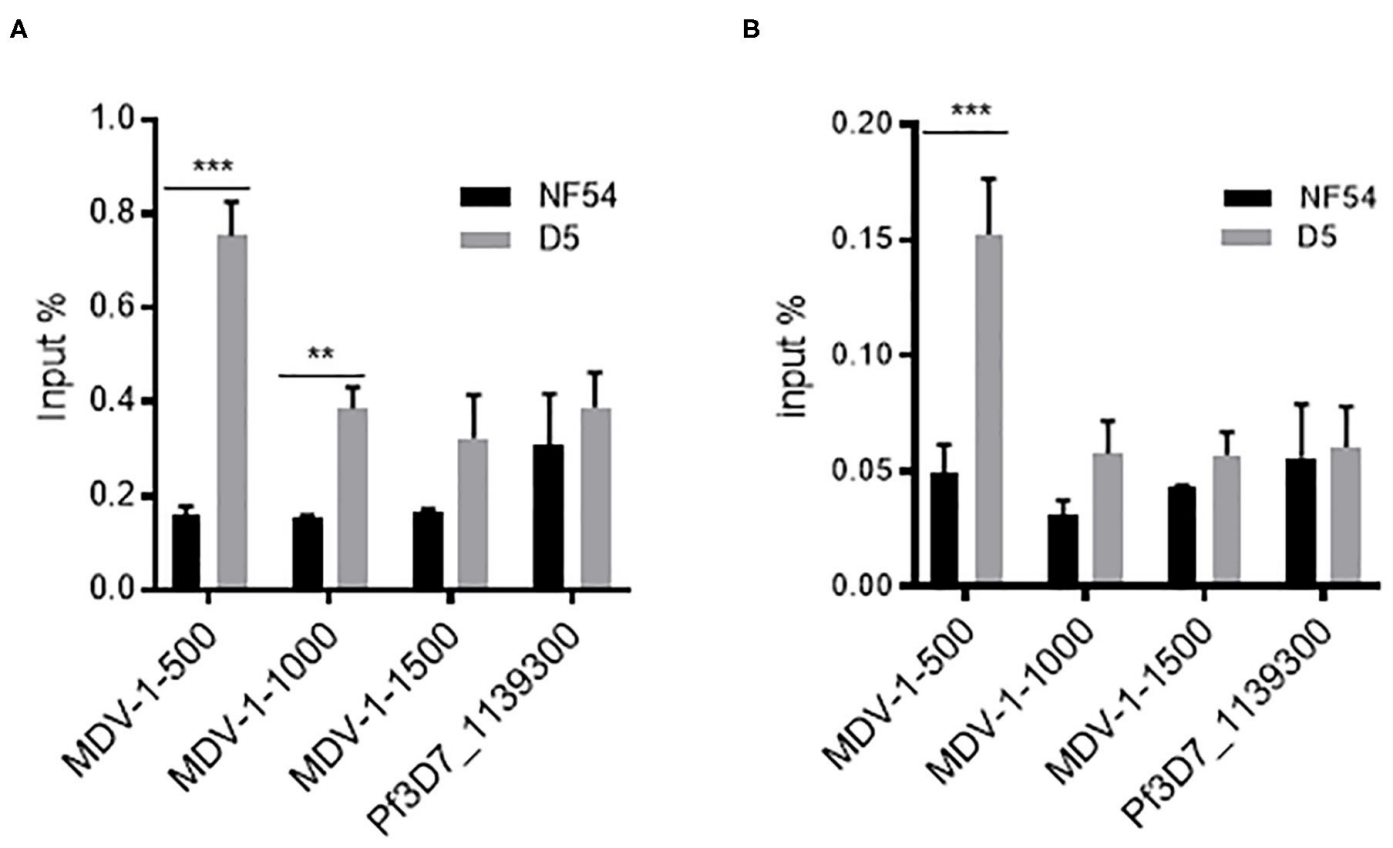
ChIP-qPCR analysis of PfAP2-G2 binding to the promoter region of *PfMDV-1* at ring stage **(A)** and schizont stage **(B)**. MDV1-500, MDV1-1000, and MDV1-1500 indicate primers that are located at about 500 bp, 1,000 bp, 1,500 bp before the start codon of *PfMDV-1*, respectively. TSS of *PfMDV-1* is mapped to about 500 bp upstream of the start codon. *Pf3D7_1139300* was used as a negative control. Primers for ChIP-qPCR are shown in Supplementary Table 1. The experiment was repeated three times. All error bars indicate SD. **P < 0.01; ***P < 0.001.

Since *PfMDV-1* was decreased both in the ring and schizont stages after *PfAP2-G2* deletion, we suspected that *PfMDV-1* may be one of the targets of PfAP2-G2. To test this hypothesis, we evaluated whether PfAP2-G2 is targeted to the *PfMDV-1* gene. Both D5 and NF54 parasites were subjected to ChIP by using the mouse anti-Flag antibody at the ring and schizont stages. qPCR analysis using primer pairs targeting to different upstream regions of the *PfMDV-1* gene showed significant enrichment of the PfAP2-G2 protein at around 500 bp upstream of the start codon, which corresponds approximately to the transcriptional start site (TSS) of *PfMDV-1* at both ring and schizont stages (Figures 4A,B). This result suggests that *PfMDV-1* is one of the targets of PfAP2-G2 in the asexual stage.

## Discussion

In this study, we showed that PfAP2-G2, whose *P. berghei* ortholog is downstream in a transcriptional cascade initiated by PbAP2-G (Sinha et al., 2014), played a critical role in the production and maturation of gametocytes. *PfAP2-G2* was highly expressed in the ring stage and localized in the nuclei of early-stage parasites. Deletion of *PfAP2-G2* reduced gametocyte production and resulted in the nearly complete (>95%) loss of mature gametocytes, especially male gametocytes. In addition, we found that *PfAP2-G2* deletion resulted in down-regulation of *PfMDV-1* in both ring stage and schizont stage. We provided further evidence to show that the *PfMDV-1* was a target of PfAP2-G2 in the asexual stage.

Consistent with the previous findings in *P. berghei* (Sinha et al., 2014; Yuda et al., 2015), deletion of *PfAP2-G2* could significantly reduce the number of mature gametocytes, which may be the consequence of PfAP2-G2 regulating the expression of *PfMDV-1*. PfMDV-1 plays an important role in maintaining membrane structures which are essential for gametocyte maturation in erythrocytes. Disruption of *PfMDV-1* results in a dramatic reduction of mature gametocytes, especially functional male gametocytes, with the majority of sexually committed parasites developmentally arrested at stage I (Guinet et al., 1996; Furuya et al., 2005). We also found that the deletion of *PfAP2-G2* resulted in the reduction of stage I gametocytes, especially male gametocytes. However, given that *PfMDV-1* is only one of the genes whose expressed is decreased after *PfAP2-G2* deletion, the defect in sexual development may also involve other gametocyte-specific genes. Due to a ~95% reduction in gametocytemia was happened in the *PfAP2-G2(-)* parasites, it is hardly for us to collect the gametocyte samples for RNA-seq, so, we decided to study the mechanisms of PfAP2-G2 in the asexual stage and found that PfAP2-G2 regulated the formation of gametocyte by directly regulate the expression of *PfMDV-1* in ring stage and schizont stage. Simultaneously, the RT-qPCR result of *PfAP2-G2* found that it also highly expressed in gametocyte stage, means PfAP2-G2 may also functionating at gametocyte stage. Recently, Singh et al. (2020) demonstrated that PfAP2-G2 can bound the promoters of genes expressed at several different stages of the parasite life cycle, as well as gene body of genes which correlates with the location of H3K36me3 and several other histone modifications. All these results demonstrated that PfAP2-G2 may have different function in the asexual stage and gametocyte stage, and the function of it in gametocyte stage would be worth to study in the future.

AP2-G2 has been identified as a transcriptional repressor that induces genome-wide transcriptional repression in early gametocytes and contributes to dramatic alterations of cell fate at this stage in *P. berghe*i (Yuda et al., 2015). Similarly, Singh et al. (2020) collected the samples in stage and gametocyte stage III for CHIP-seq, suggesting that PfAP2-G2 acted as a repressor and affected the transcription by interacting with other proteins. However, we found that *PfMDV-1* was down-regulated in *PfAP2-G2(-)* parasites. Here, we propose two hypotheses to explain this phenomenon. First, AP2-G2 may induce genome-wide transcriptional repression in *P. falciparum* throughout the life cycle of parasites. *PfMDV-1* may be up-regulated in gametocytes after the point at which PfAP2-G2 is required for continued development. In that case, *PfAP2-G2* knockout cultures would lack the background levels of gametocytes expressing these genes that are present in wild-type cultures. Second, we found that compared with the NF54 parasites, the deletion of *PfAP2-G2* resulted in significantly more genes up-regulated (113 genes) in the ring stage. Contrasting, in schizont stage, more genes were down-regulated (102 genes). As a result, we speculate that PfAP2-G2 may function as a transcriptional repressor for some of the genes at ring stage, but not all stages. The function of PfAP2-G2 is complicated and so, worth further examination.

In *P. berghei*, ChIP-seq analysis has revealed that targets of PbAP2-G2 mainly included genes that were required for asexual proliferation, whereas gametocyte-specific genes were not considered (Yuda et al., 2015). Targets of AP2-G2 appear to be highly divergent in *P. falciparum* and *P. berghei*, although the phenotypes are similar (Campbell et al., 2010). In this study, RNA-seq analysis showed that a limited number of genes with defined biological functions were up or down regulated in *PfAP2-G2(-)* parasites. Among genes identified as regulons of gametocytes in *P. falciparum* (Josling and Llinas, 2015), we only found reduced expression of *PfMDV-1* in *PfAP2-G2(-)* parasites. Thus, we speculated that *PfMDV-1* might be regulated by PfAP2-G2 directly, as ChIP-qPCR analysis identified binding of PfAP2-G2 to the putative promoter of *PfMDV-1*. We also detected other genes which might be gametocyte-specific genes and were down-regulated at ring stage simultaneously. However, we found that CHIP-qPCR analysis using primer pairs targeting to different upstream regions of these genes showed no significant enrichment of the PfAP2-G2 protein. This may be because of the fact that these genes are important to gametocytes development, loss of *PfAP2-G2* would cause the decrease of gametocytes so that the expression of these genes descends (Supplementary Figure 3). Interestingly, the expression of *PfAP2-G* was also down-regulated in *PfAP2-G2(-)* parasites at the schizont stage, which partially explains the reduced gametocytogenesis phenotype. The mechanism of ApiAP2 regulators may be different in Plasmodium species. We speculate that PfAP2-G2 may mainly execute its role at the upstream to regulate *PfAP2-G*, whose expression is the initial of sexual commitment through the next cycle conversion pathway (NCC pathway) (Bancells et al., 2019). In addition, it is possible that PfAP2-G2 is at downstream. It is also possible that there is a feedback regulation and synergy affection between PfAP2-G2 and PfAP2-G. However, we do not know whether PfAP2-G2 affects the expression of *PfAP2-G* directly or indirectly given that PfAP2-G also regulate invasion-related genes expressed in schizont. It is worth mentioning that the expression of *PfAP2-G2* was up-regulated with the primers located close to the start codon, which is in accord with RNA-seq analysis (Supplementary Figure 4A). However, we found that there was nearly no expression of *PfAP2-G2* when the primers located in the knockout domain (Supplementary Figure 4B). In our opinion, this is because we don't knock out the whole of *PfAP2-G2*, but part of it containing the domain, which is shown on PlasmoDB and previous report (Campbell et al., 2010). Therefore, up-regulation of *PfAP2-G2* in *PfAP2-G2(-)* ring stages might be due to the transcriptional compensatory mechanism. Importantly, we confirm that *PfAP2-G2* can't be translated into protein successfully.

In conclusion, we have shown that PfAP2-G2 is highly expressed in the ring stage and localized in the nuclei of all stages of asexual parasites. More importantly, it plays an important role in the production and maturation of gametocytes, especially the male gametocytes. RNA-seq and ChIP-qPCR analyses suggest that PfAP2-G2 targets the *PfMDV-1* gene and regulates its expression in the asexual stage.

## Data Availability Statement

The data presented in the study are deposited in the NCBI repository, accession number PRJNA686696.

## Author Contributions

YX, JG, YMC, and ZH conceived and designed experiments. YX performed the majority of the experiments. DQ, YW, YB, and YXC collected the samples and conducted the bioinformatics analysis. YX, DQ, YW, YB, JG, YXC, ZH, LC, and YMC wrote the manuscript. All authors discussed and edited the manuscript.

## Conflict of Interest

The authors declare that the research was conducted in the absence of any commercial or financial relationships that could be construed as a potential conflict of interest.
