# Supplementary material for: PfAP2-G2 Is Associated to Production and Maturation of Gametocytes in Plasmodium falciparum via Regulating the Expression of PfMDV-1
Source: Front Microbiol. 2021 Jan 18;11:631444. doi: 10.3389/fmicb.2020.631444 (PMC7848025; doi:10.3389/fmicb.2020.631444)
Supplement: Supplementary file 4 [file Presentation_1.PPTX]

## Slide 1
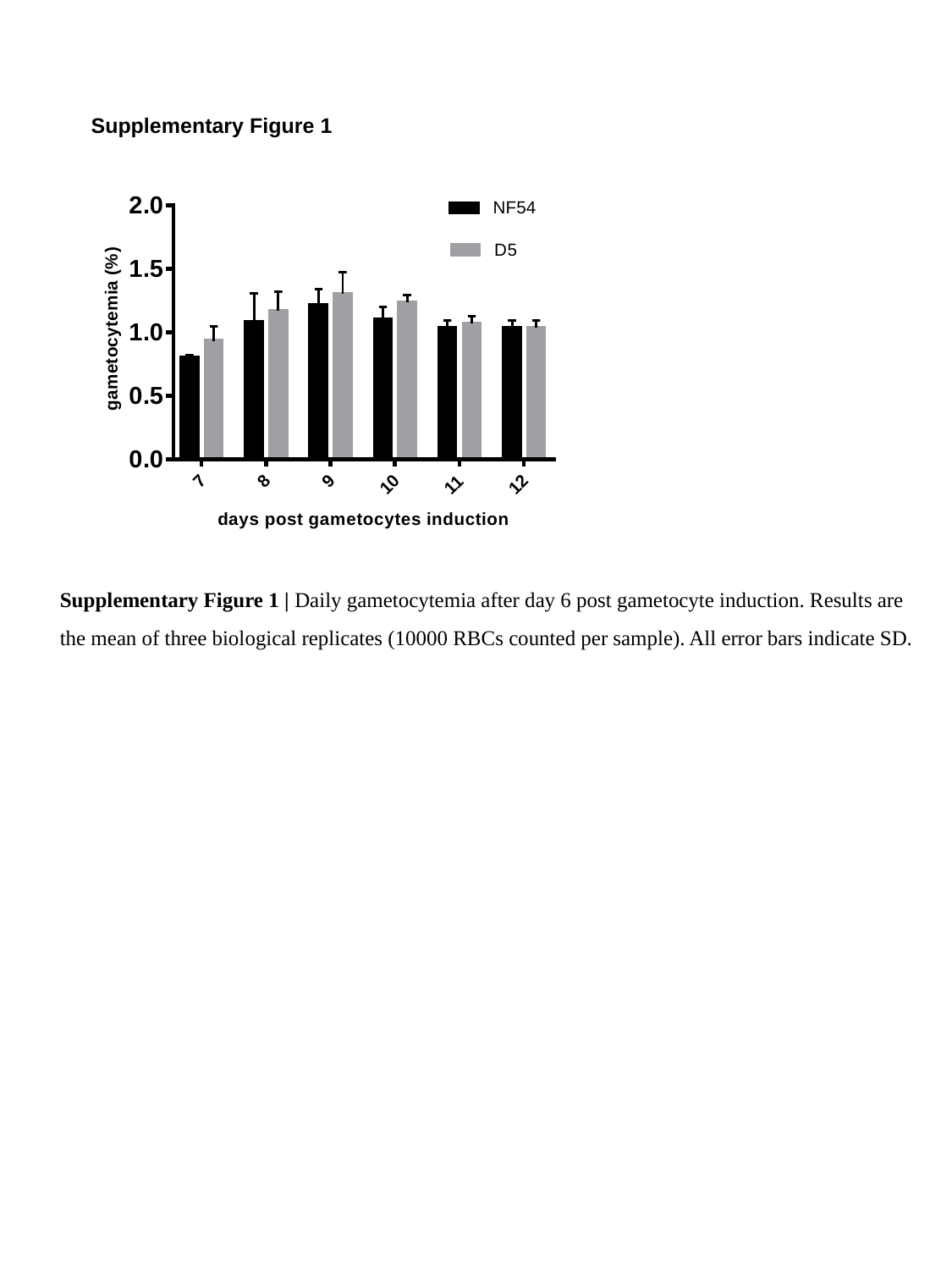

Supplementary Figure 1
Supplementary Figure 1 | Daily gametocytemia after day 6 post gametocyte induction. Results are the mean of three biological replicates (10000 RBCs counted per sample). All error bars indicate SD.

## Slide 2
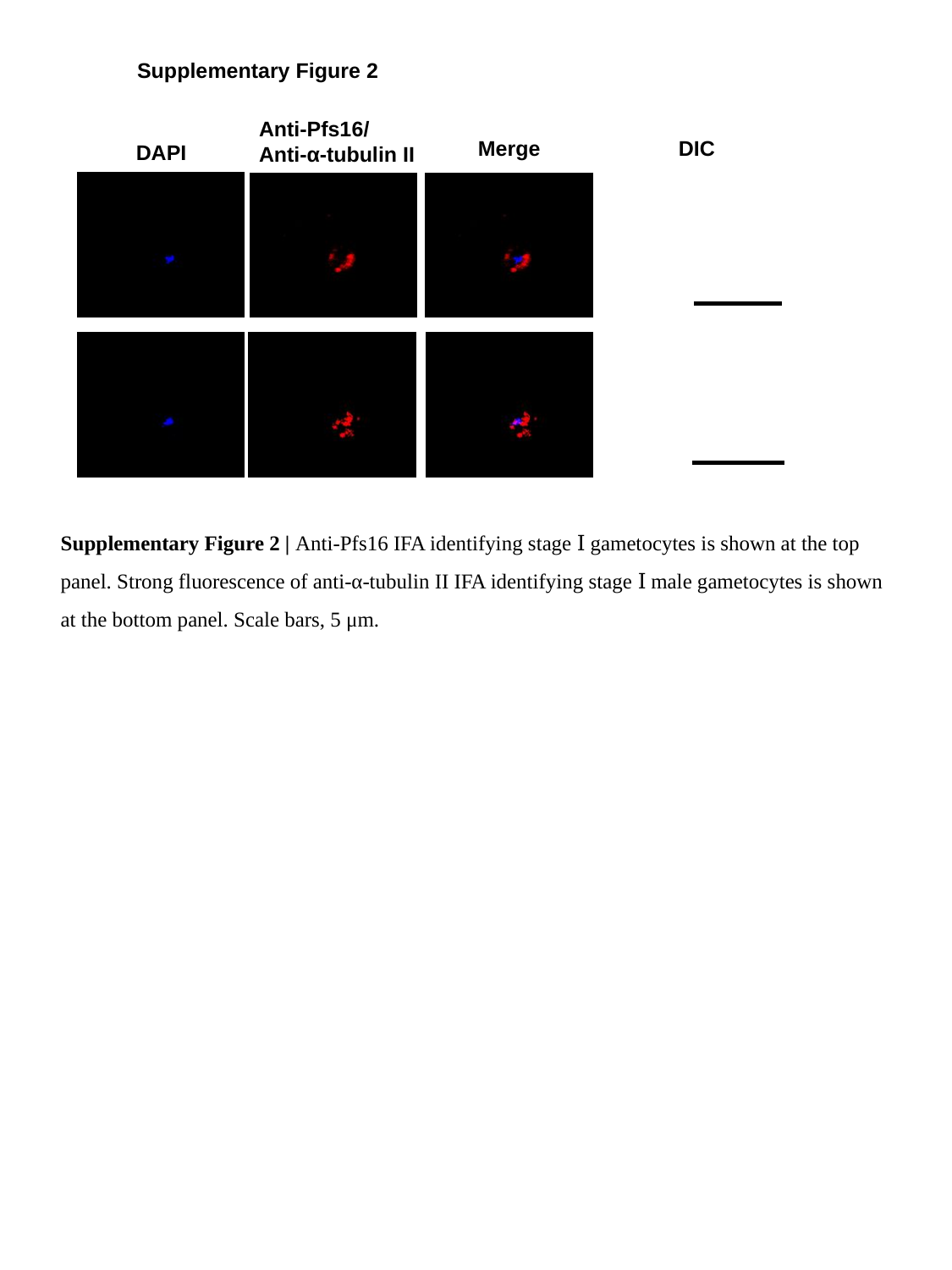

Supplementary Figure 2
Anti-Pfs16/
Anti-α-tubulin II
Merge
DIC
DAPI
5μm
5μm
Supplementary Figure 2 | Anti-Pfs16 IFA identifying stage Ⅰ gametocytes is shown at the top panel. Strong fluorescence of anti-α-tubulin II IFA identifying stage Ⅰ male gametocytes is shown at the bottom panel. Scale bars, 5 μm.

## Slide 3
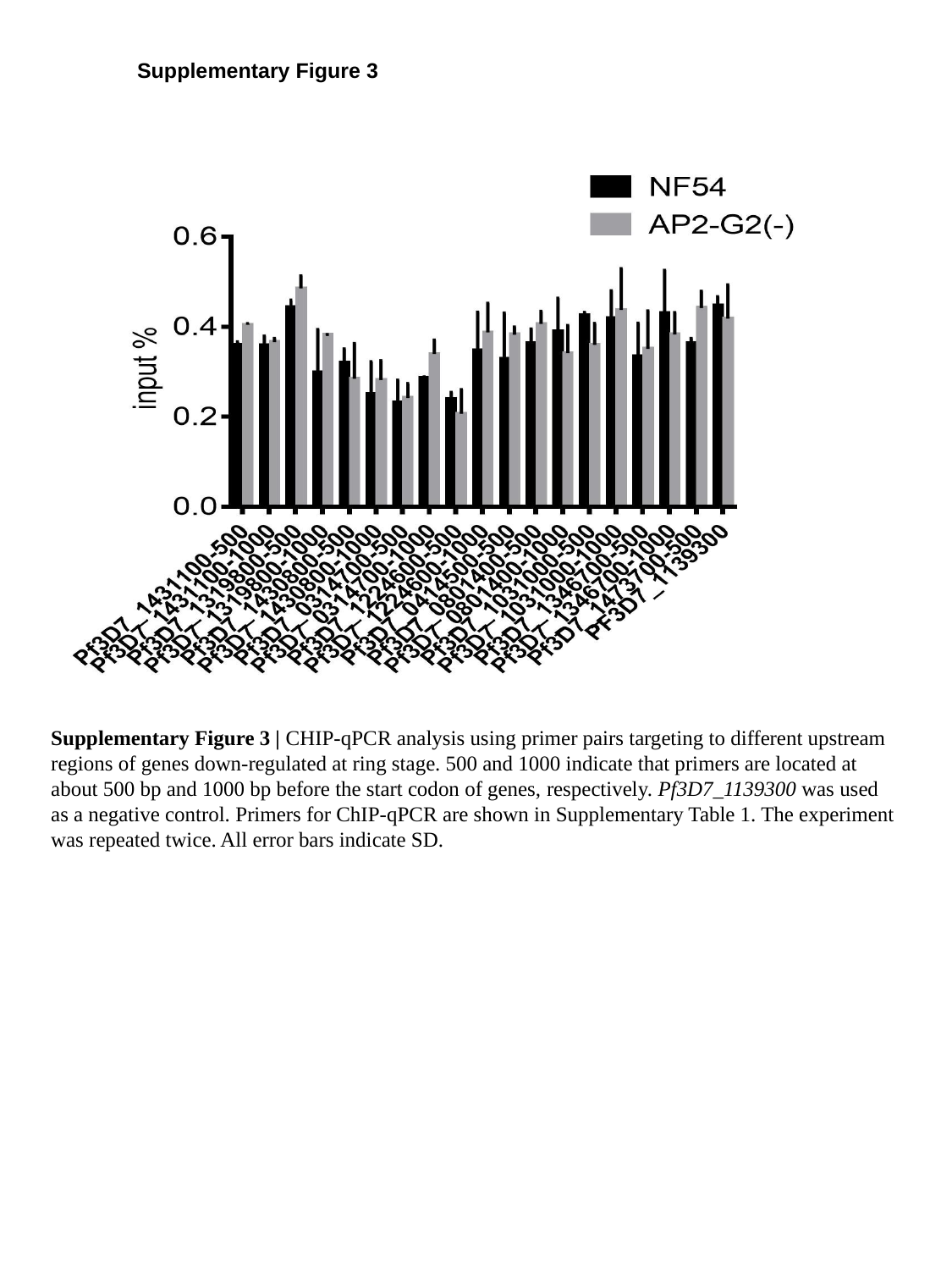

Supplementary Figure 3
Supplementary Figure 3 | CHIP-qPCR analysis using primer pairs targeting to different upstream regions of genes down-regulated at ring stage. 500 and 1000 indicate that primers are located at about 500 bp and 1000 bp before the start codon of genes, respectively. Pf3D7_1139300 was used as a negative control. Primers for ChIP-qPCR are shown in Supplementary Table 1. The experiment was repeated twice. All error bars indicate SD.

## Slide 4
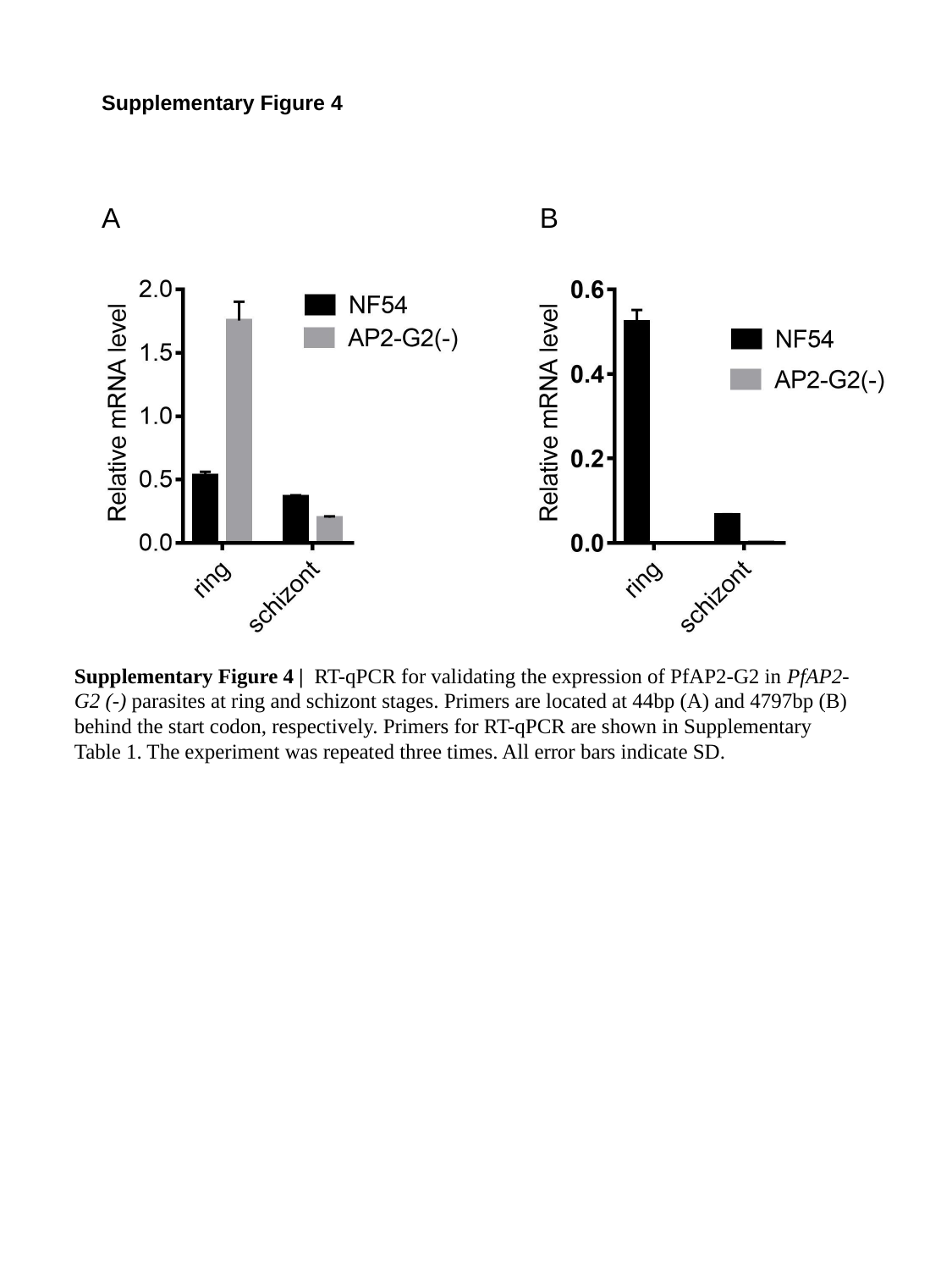

Supplementary Figure 4
A
B
Supplementary Figure 4 | RT-qPCR for validating the expression of PfAP2-G2 in PfAP2-G2 (-) parasites at ring and schizont stages. Primers are located at 44bp (A) and 4797bp (B) behind the start codon, respectively. Primers for RT-qPCR are shown in Supplementary Table 1. The experiment was repeated three times. All error bars indicate SD.
